# Cerebral Abscess Caused by Histoplasma capsulatum in an Immunocompetent Patient: A Case Report

**DOI:** 10.7759/cureus.86503

**Published:** 2025-06-21

**Authors:** Juliana S Zanotti, Fernanda P Rodrigues, Alex Michel Daoud, João Luiz Vitorino Araujo, Jean Gonçalves de Oliveira

**Affiliations:** 1 Neurological Surgery, Irmandade da Santa Casa de Misericórdia de São Paulo, São Paulo, BRA

**Keywords:** brain abscess, case report, cns histoplasmosis, fungal infection, histoplasma capsulatum, immunocompetent, neurosurgery

## Abstract

Central nervous system (CNS) histoplasmosis is a rare manifestation of Histoplasma capsulatum infection and is typically associated with disseminated disease in immunocompromised individuals. However, isolated CNS involvement in immunocompetent patients remains uncommon and poses a diagnostic challenge due to its nonspecific clinical presentation. We report the case of a 44-year-old immunocompetent male patient with a history of intravenous drug use who presented with seizures and progressive headache. Brain imaging revealed a left frontal ring-enhancing lesion with midline shift and surrounding edema. Despite negative systemic workup and serologies, the patient underwent surgical drainage of the abscess, and histopathological analysis confirmed infection by H. capsulatum. He was treated with liposomal amphotericin B for 50 days, resulting in full clinical recovery without neurological deficits. This case highlights the importance of including histoplasmosis in the differential diagnosis of cerebral abscesses, even in immunocompetent individuals from endemic areas, especially when noninvasive diagnostics are inconclusive. Early surgical intervention combined with appropriate antifungal therapy can lead to excellent outcomes.

## Introduction

Brain abscesses are focal infections within the brain parenchyma, often secondary to neurosurgical procedures, trauma, or contiguous/systemic infections [1]. They represent approximately 8% of the intracranial masses in low-income countries and 1-2% in high-income regions, with an overall incidence of four cases per million people. Differential diagnoses include brain tumors, mycotic aneurysms, and septic dural sinus thrombosis [2].

Fungal brain abscesses can occur in both immunocompetent and immunocompromised individuals. In immunocompetent patients, Candida spp. and mucormycosis are the most frequent agents, particularly among intravenous drug users [1].

Central nervous system (CNS) involvement by Histoplasma capsulatum occurs in 5-10% of patients with disseminated histoplasmosis [3], often without pulmonary involvement [4]. The most common CNS manifestations are chronic meningitis, focal lesions, stroke-like syndromes, and encephalitis [3]. A brain abscess due to Histoplasma is rare and typically associated with disseminated disease.

Here, we report a rare case of a brain abscess caused by H. capsulatum in an immunocompetent patient with no evidence of systemic histoplasmosis. The patient responded favorably to surgical drainage followed by targeted antifungal therapy. This case underscores the importance of considering Histoplasma infection in patients from endemic regions, even in the absence of systemic findings.

## Case presentation

A 44-year-old male patient with a history of intravenous drug use and a dental abscess two years earlier, presented to the emergency department with a two-year history of headaches and seizures, acutely worsening over the previous two days. Neurological examination revealed right-sided central facial palsy and dysarthric speech. No other focal deficits were noted. He was otherwise functionally independent at baseline.

**Figure 1:**
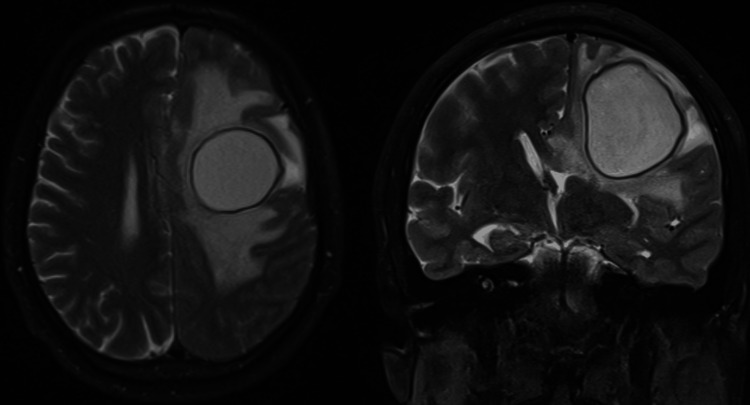
T2 MRI showing the hyperintense center, hypointense capsule, and vasogenic edema surrounding the abscess

A non-contrast head CT revealed a hypodense lesion with a hyperdense ring in the left frontal lobe (4.2 × 3.8 cm), causing a 1 cm midline shift and significant vasogenic edema. An MRI showed a rounded intra-axial lesion in the left frontal centrum semiovale with restricted diffusion, surrounded by vasogenic edema extending to the subinsular region and corpus callosum (Figure 1).

Empirical antibiotic therapy was initiated. Chest and abdominal contrast-enhanced CT scans revealed an 11 cm cavitary lesion, initially suggestive of tuberculosis. Serologies for HIV, hepatitis B, syphilis, Paracoccidioides, and H. capsulatum were negative. Bronchoalveolar lavage cultures and lung biopsy were negative for mycobacteria, fungi, and acid-fast bacilli.

**Figure 2:**
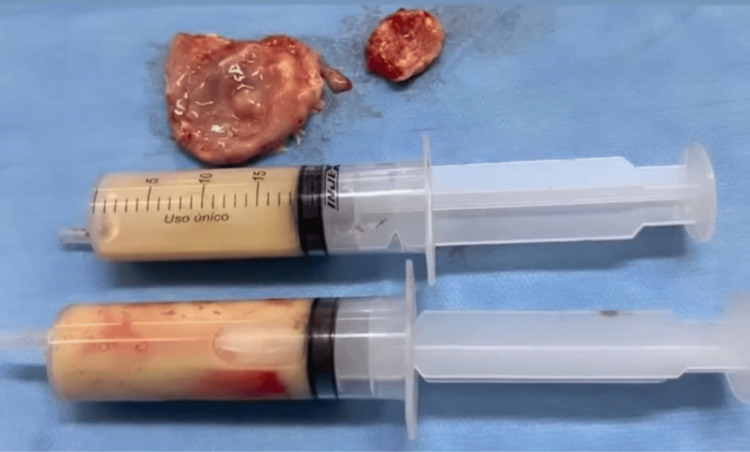
Purulent material and the excised capsule of the abscess

On the fifth day of hospitalization, the patient underwent left frontoparietal craniotomy with ultrasound-guided abscess drainage. A transverse incision over the left coronal suture was followed by microsurgical aspiration of 37 mL of purulent material and total capsule excision. The specimens were sent for histopathology and microbiology (Figure 2).

Postoperatively, the patient was extubated in the OR and transferred to the ICU, with immediate resolution of the headache.

Histopathology revealed a fungal abscess with morphology consistent with H. capsulatum. He received liposomal amphotericin B for 50 days and was discharged with full clinical recovery. Immunological workup showed no evidence of immunodeficiency. The patient remains under outpatient follow-up without neurological sequelae.

## Discussion

CNS histoplasmosis is an uncommon manifestation of H. capsulatum infection, most frequently observed in immunocompromised individuals or in association with disseminated disease. However, an increasing number of cases have been reported in immunocompetent patients, particularly in endemic areas, which poses diagnostic challenges and often results in delayed treatment [3,5].

In this report, we describe a rare case of an isolated brain abscess caused by H. capsulatum in an immunocompetent individual, without any systemic signs of histoplasmosis. Although CNS involvement typically occurs in 5-10% of disseminated cases, up to 29% of patients may be immunocompetent, and isolated CNS presentations are increasingly recognized [3]. The pathogenesis may involve hematogenous spread from a subclinical pulmonary focus or, as hypothesized in this patient, direct seeding from prior injection drug use [6].

The clinical spectrum of CNS histoplasmosis is broad, with headache, altered mental status, and focal neurological signs being common manifestations [3,5]. Our patient presented with seizures and right-sided central facial palsy. While chronic meningitis is the most frequent form of presentation, focal parenchymal lesions such as histoplasmomas or abscesses have been reported, albeit rarely [5,6].

MRI findings of ring-enhancing lesions with restricted diffusion are characteristic of fungal abscesses and were present in this case. Similar imaging findings have been reported in other cases of CNS histoplasmosis, particularly those with abscess formation [6]. However, these radiological features are not specific, and a definitive diagnosis requires histopathological or microbiological confirmation.

Diagnosis is often delayed due to the low sensitivity of CSF cultures and the nonspecific nature of clinical and imaging findings. Combined testing of CSF for antigen and antibody has shown diagnostic yield in up to 75-80% of cases and is currently recommended as the initial non-invasive diagnostic approach [3,6]. In the present case, however, definitive diagnosis was achieved through surgical resection and histopathological identification of the fungal elements.

The mainstay of treatment is antifungal therapy with liposomal amphotericin B followed by an oral azole. According to Infectious Diseases Society of America (IDSA) guidelines, at least four to six weeks of amphotericin B, followed by 12 months of itraconazole, is recommended [6]. Our patient was treated with liposomal amphotericin B for 50 days and demonstrated full clinical recovery without sequelae. Long-term monitoring is essential due to the risk of relapse, particularly in cases of incomplete treatment or underlying immunodeficiency [3].

This case highlights the importance of considering H. capsulatum in the differential diagnosis of brain abscesses in patients from endemic regions, even in the absence of overt immunosuppression or systemic disease [5-8].

## Conclusions

This case highlights the rare occurrence of isolated CNS histoplasmosis presenting as a brain abscess in an immunocompetent patient without evidence of disseminated disease. Although uncommon, similar presentations have been increasingly recognized, particularly in endemic regions. It underscores the importance of considering H. capsulatum in the differential diagnosis of intracranial ring-enhancing lesions, even in immunocompetent hosts. Prompt surgical intervention combined with antifungal therapy, as recommended by the current guidelines, can result in favorable outcomes. Therefore, clinicians should maintain a high index of suspicion and pursue early histopathological confirmation when non-invasive diagnostics are inconclusive.
